# Multi-step biosynthesis of the biodegradable polyester monomer 2-pyrone-4,6-dicarboxylic acid from glucose

**DOI:** 10.1186/s13068-023-02350-y

**Published:** 2023-06-01

**Authors:** Dan Zhou, Fengli Wu, Yanfeng Peng, Muneer Ahmed Qazi, Ruosong Li, Yongzhong Wang, Qinhong Wang

**Affiliations:** 1 Key Laboratory of Biorheological Science and Technology (Chongqing University), Ministry of Education, College of Bioengineering, Chongqing University, Chongqing, 400030 China; 2 Tianjin Institute of Industrial Biotechnology, Chinese Academy of Sciences, Tianjin, 300308 China; 3 National Center of Technology Innovation for Synthetic Biology, Tianjin, 300308 China; 4 Institute of Microbiology, Faculty of Natural Science, Shah Abdul Latif University, Khairpur, 66020 Sindh Pakistan; 5 College of Biotechnology, Tianjin University of Science & Technology, Tianjin, 300457 China

**Keywords:** Aromatic compounds, Bioconversion, Biodegradable plastics, 3-Dehydroshikimate dehydratase, Protocatechuate 4,5-dioxygenase, CHMS dehydrogenase, Protocatechuate 4,5-cleavage pathway, *Escherichia coli*, Whole-cell biocatalyst

## Abstract

**Background:**

2-Pyrone-4,6-dicarboxylic acid (PDC), a chemically stable pseudoaromatic dicarboxylic acid, represents a promising building block for the manufacture of biodegradable polyesters. Microbial production of PDC has been extensively investigated, but low titers and yields have limited industrial applications.

**Results:**

In this study, a multi-step biosynthesis strategy for the microbial production of PDC was demonstrated using engineered *Escherichia coli* whole-cell biocatalysts. The PDC biosynthetic pathway was first divided into three synthetic modules, namely the 3-dehydroshikimic acid (DHS) module, the protocatechuic acid (PCA) module and the PDC module. Several effective enzymes, including 3-dehydroshikimate dehydratase for the PCA module as well as protocatechuate 4,5-dioxygenase and 4-carboxy-2-hydroxymuconate-6-semialdehyde dehydrogenase for the PDC module were isolated and characterized. Then, the highly efficient whole-cell bioconversion systems for producing PCA and PDC were constructed and optimized, respectively. Finally, the efficient multi-step biosynthesis of PDC from glucose was achieved by smoothly integrating the above three biosynthetic modules, resulting in a final titer of 49.18 g/L with an overall 27.2% molar yield, which represented the highest titer for PDC production from glucose reported to date.

**Conclusions:**

This study lays the foundation for the microbial production of PDC, including one-step de novo biosynthesis from glucose as well as the microbial transformation of monoaromatics.

**Supplementary Information:**

The online version contains supplementary material available at 10.1186/s13068-023-02350-y.

## Background

Polymers (such as polyesters and polyamides) and their precursors account for the largest market share (> $500 billion) of all currently manufactured chemical products [1]. Through a polycondensation approach between di- or polyfunctional carboxylic acids and di- or polyfunctional alcohols, a variety of polyesters can be produced, which is the most applied method [2]. Poly(alkylene terephthalate)s, such as poly(ethylene terephthalate) (PET) and poly(butylene terephthalate) (PBT), represent one of the most frequently used polymer classes worldwide [3, 4]. However, at the same time, more than 400 million tons of plastic waste are produced each year, most of which are landfilled or discarded, while the rest is incinerated, thereby causing serious environmental contamination [4, 5]. Therefore, the development of biodegradable polyesters as well as the bioconversion of plastic waste represent two efficient strategies for a circular economy whereby waste prevention and recycling are encouraged. So far, a number of biodegradable polymers, such as poly(lactic acid) (PLA) [6, 7], poly(butylene succinate) (PBS) [8, 9] and polyhydroxyalkanoates (PHAs) [7] have been applied in many fields, but they all belong to aliphatic polymers that lack rigidity due to their innate structure, thus leading to weaker thermomechanical properties compared with oil-based PET [9–12].

2-Pyrone-4,6-dicarboxylic acid (PDC) is a novel and chemically stable dibasic acid that consists of a polar pseudoaromatic pyran ring as well as two carboxyl groups [13, 14]. Given its structural similarity with terephthalic acid (TPA), PDC can also be polymerized with various diols in the presence of appropriate catalysts to produce the corresponding polyesters. At the same time, due to the pseudoaromatic nature of PDC, its derived polyesters possess many excellent properties, such as better thermal stability [13, 15, 16], enhanced biodegradability [17], high elasticity [17], strong adhering properties [15] and a higher Young’s modulus [13], amongst others, compared with PET. Therefore, PDC is considered to be a promising alternative monomer to TPA for the preparation of synthetic plastics.

PDC is naturally produced by some microbes during the biodegradation of lignin-derived aromatic compounds [18–21]. So far, the microbial synthesis of PDC has been achieved using different approaches, such as whole-cell bioconversion of pure monoaromatics or lignin-derived ones as well as de novo biosynthesis from glucose. In this context, protocatechuate 4,5-dioxygenase (an *α*_2_*β*_2_ tetramer, LigAB) and 4-carboxy-2-hydroxymuconate-6-semialdehyde (CHMS) dehydrogenase (LigC), within the protocatechuate 4,5-cleavage pathway, represent two indispensable enzymes for the bioconversion of protocatechuic acid (PCA) into PDC [18, 22]. The *Pseudomonas putida* PpY1100/pDVABC cells, engineered to co-express the *ligAB* and *ligC* genes derived from *Sphingomonas paucimobilis* SYK-6, were employed to perform whole-cell bioconversion, and approximately 11 g/L PDC was stably produced from PCA within 36 h with a jar-fermentor system [22]. Subsequently, the microbial conversion of lignin-derived monoaromatics (vanillin and vanillic acid) into PDC (~ 50 mM) by *P. putida* PpY1100/pVapoligVABC whole-cells was also realized [23]. The *ligAB* and *ligC* genes were constitutively overexpressed in *P. putida* KT2440 strain which also harbored a double deletion of the *pcaGH* and *crc* genes, and these promoted PDC accumulation up to 22.7 g/L during fed-batch fermentation with *p*-coumaric acid and/or ferulic acid as substrates [16]. Perez et al. engineered *Novosphingobium aromaticivorans* DSM12444 strain, an organism known to degrade aromatic compounds, to accumulate PDC by deleting the *ligI* and *desCD* genes in the degradation pathway. The resulting strain was then able to convert vanillin and vanillic acid into 4.9 g/L of PDC within 48 h using a pH-controlled fed-batch conversion approach [20]. Besides, microalgae hydrolysate and PET-derived TPA were also used as substrates for the microbial production of PDC, but the titer or yield was relatively low [24, 25]. De novo biosynthesis of PDC from glucose was first realized in recombinant *Escherichia coli* cells that co-expressed the essential genes in the shikimate pathway as well as in the protocatechuate 4,5-cleavage pathway [26]. Luo et al. subsequently constructed a series of PDC-overproducing *E. coli* strains using stepwise rational metabolic engineering strategies. The best-performing engineered strain produced 16.72 g/L of PDC with an overall productivity of 0.172 g/L/h from glucose by fed-batch fermentation [27].

The low titer or yield of PDC in the above reports makes it difficult for the compound to become an economically competitive monomer for the bio-based polyester industry. It was, therefore, speculated that the following factors could be limiting the titer or yield of PDC: (i) The whole-cell bioconversion systems or conditions were not optimal; (ii) The enzymic activities were low; (iii) High concentrations of the aromatic compounds were cytotoxic to the cells or exhibited strong feedback inhibition on enzymatic reactions; (iv) The metabolic flux of the shikimate pathway was too low to supply enough precursors for the biosynthesis of PDC. In this study, based on well-characterized and effective enzymes as well as established highly efficient whole-cell bioconversion systems, an efficient method for the biosynthesis of PDC from glucose with a higher titer was finally developed.

## Materials and methods

### Strains, plasmids and chemicals

All the strains and plasmids used in this study are listed in Additional file 1: Table S1. *E. coli* DH5α was employed for gene cloning and plasmid propagation, and *E. coli* BL21(DE3) and its derivatives were used for recombinant protein expression and whole-cell biocatalyst preparation. Glucose was purchased from Sangon Biotech Co., Ltd. (Shanghai, China), 3-dehydroshikimic acid (DHS, purity ≥ 95%) was purchased from Sigma–Aldrich (Shanghai, China), and gallic acid (GA, purity > 98%), PCA (purity > 98%) and PDC (purity > 97%) were purchased from Bide Pharmatech Ltd. (Shanghai, China).

### Construction of phylogenetic tree

The protein sequences of protocatechuate 4,5-dioxygenase and CHMS dehydrogenase were all retrieved from the National Center for Biotechnology Information (NCBI) database (https://www.ncbi.nlm.nih.gov) and were listed in Additional file 1: Table S2. The protein sequences of the protocatechuate 4,5-dioxygenase *α* subunit were analyzed using the phylogenetic package MEGA X [28]. The phylogenetic tree was constructed using the neighbor-joining (NJ) method with 1000 bootstrap replications.

### Plasmid construction

To construct the expression plasmids, the genes of *ligAB* and *ligC* derived from different organisms (Additional file 1: Table S2) were codon-optimized for expression in *E. coli*, and then cloned into the pRSFDuet-1 plasmid at the NcoI–HindIII and NdeI–XhoI sites, respectively. The coding sequences of *ligA* and *ligB* were separated by the ribosome binding site (RBS) sequence (5′-GGATCCGAAGGAGATATACC-3′). The genes encoding 3-dehydroshikimate dehydratases (GenBank: WP_015958588.1, ABY99959.1, AAC37159.1, KJL23089.1 and OES41209.1) derived from different organisms were also codon-optimized, and then cloned into the pET-30a plasmid at the NdeI–XhoI site.

### Media and growth conditions

*Escherichia coli* cells were routinely cultivated in liquid Luria–Bertani (LB) medium (10 g/L tryptone, 5 g/L yeast extract, 10 g/L NaCl) or on LB agar plates at 37 ℃. The medium was supplemented with 50 μg/mL kanamycin when needed.

For protein expression in shake flasks, a single colony was inoculated into 5 mL of LB medium and cultured overnight at 37 °C and 250 rpm. The overnight seed culture was then inoculated into a 500 mL shake flask containing 100 mL LB medium at a ratio of 1:100 and incubated at 37 °C and 250 rpm. When the cell density (OD_600_) reached 0.6, 0.2 mM isopropyl-β-d-thiogalactopyranoside (IPTG) was added into the shake flask to induce protein expression. After induction at 28 °C for an additional 10 h, the cells were harvested by centrifugation and directly used as whole-cell biocatalysts.

For protein expression on a large scale, fed-batch fermentation was conducted in a 5-L bioreactor (BIOTECH-5BG, Bxbio, China). A single colony was inoculated into a falcon tube containing 5 mL LB medium and cultured overnight at 37 °C and 250 rpm. The overnight seed was then inoculated into 200 mL LB medium at a ratio of 1:100 and incubated at 37 °C and 250 rpm for 10–12 h. After this, the seed culture was transferred into 2.3 L fermentation medium supplemented with 50 μg/mL kanamycin and cultivated at 37 °C. The agitation, air supplementation and feed rate were changed to maintain the dissolved oxygen (DO) concentration above 30% saturation. The pH was controlled at 7.0 using 25% (w/v) NH_3_·H_2_O. The DO-stat feeding strategy was employed to supply feeding medium into the bioreactor. When the OD_600_ reached 30–40, the culture was cooled to 30 °C, and then 0.5 mM IPTG was added into the bioreactor to induce protein expression. Samples were collected every 2 h to determine the OD_600_ and residual glycerol. After induction at 30 °C for an additional 6 h, the cells were harvested by centrifugation and directly used as whole-cell biocatalysts. The fermentation medium contained 10 g/L glycerol, 24 g/L yeast extract, 12 g/L tryptone, 16.43 g/L K_2_HPO_4_·3H_2_O, and 2.31 g/L KH_2_PO_4_. The feeding medium contained 600 g/L glycerol, 57.5 g/L yeast extract, 92.5 g/L tryptone, and 10 g/L MgSO_4_·7H_2_O.

For the preparation of DHS fermentative broth, fed-batch fermentation of the WJ060 strain was conducted as previously described [29]. After fermentation, the cells were removed by centrifugation, and the supernatant with a high concentration of DHS was used as the substrate for whole-cell bioconversion.

### Whole-cell bioconversion

For bioconversion of PCA into PDC using the shake flask, a 10 mL scale reaction mixture consisting of minimal M9 medium (4.0 g/L glucose, 17.1 g/L Na_2_HPO_4_·12H_2_O, 3.0 g/L KH_2_PO_4_, 1.0 g/L NH_4_Cl, 0.5 g/L NaCl, 0.49 g/L MgSO_4_·7H_2_O, and 0.015 g/L CaCl_2_·2H_2_O, pH 6.5 or 7.0) or 100 mM sodium phosphate buffer (pH 5.0 to 8.0) or 100 mM Tris–HCl buffer (pH 7.0), 1 or 5 OD_600_ of whole-cell biocatalyst and 1 or 5 g/L PCA was conducted in a 100 mL shake flask and incubated at 37 °C, 250 rpm.

For bioconversion of PCA into PDC using the bioreactor, the reaction mixture contained 1 L M9 medium, 10 g/L PCA and 30 OD_600_ of whole-cell biocatalyst. The reaction was performed in a 5-L bioreactor (BIOTECH-5BG, Bxbio, China) at 37 °C and 500 rpm with an airflow of 2.0 vvm. The pH was maintained at 6.5 using 10 M NaOH. For supplementing PCA, approximately 10 g PCA was added into the bioreactor at one time when the DO level rose.

For bioconversion of DHS into PCA, the reaction mixture contained DHS fermentative broth and 0.1–2.0 OD_600_ of whole-cell biocatalysts. The reaction was conducted in a 100 mL shake flask or a 5-L bioreactor (BIOTECH-5BG, Bxbio, China) at 37 °C.

### Analytical methods

Cell growth was monitored by measuring the optical density at 600 nm (OD_600_) using a SP-723 spectrophotometer (Shanghai Spectrum Instruments Ltd., China). The concentrations of DHS, GA, PCA, PDC and residual glycerol or glucose were determined by high-performance liquid chromatography (HPLC) (1260 series, Agilent Technologies, USA) equipped with a Rezex™ RFQ-Fast Acid H + (8%) column (100 × 7.8 mm) (Phenomenex, USA). The culture sample was centrifuged at 12,000 rpm for 10 min to remove cells, and the supernatant was filtered using a 0.22 μm polyethersulfone (PES) membrane filter. The injection volume was 5 μL. The analysis was performed at 55 °C with a mobile phase of 5 mM H_2_SO_4_ at a flow rate of 0.6 mL/min. The diode array detector (DAD) was used to monitor the signal at 210 nm for DHS and PCA and at 313 nm for PDC. Glycerol and glucose were quantitated by the refractive index detector (RID). Data processing and statistical analysis were conducted using EXCEL software (Microsoft Office, USA).

To further confirm the production of PDC, the culture supernatant was acidified to pH 1.0 by concentrated HCl. An aliquot (300 μL) of the resulting solution was extracted twice with the same volume of ethyl acetate. The ethyl acetate layer solution was dried on a rotary evaporator. The residue was dissolved in 300 μL of pyridine and incubated with 300 μL of bistrimethylsilyl–trifluoroacetamide (BSTFA) at room temperature for 1 h to prepare trimethylsilyl derivatives. The samples were analyzed by a gas chromatography-mass spectrometry (GC–MS) system (7890A/5975C, Agilent Technologies, USA) as described previously [22].

## Results and discussion

### Establishment of a highly efficient whole-cell biocatalytic system for the conversion of PCA into PDC

**Fig. 1:**
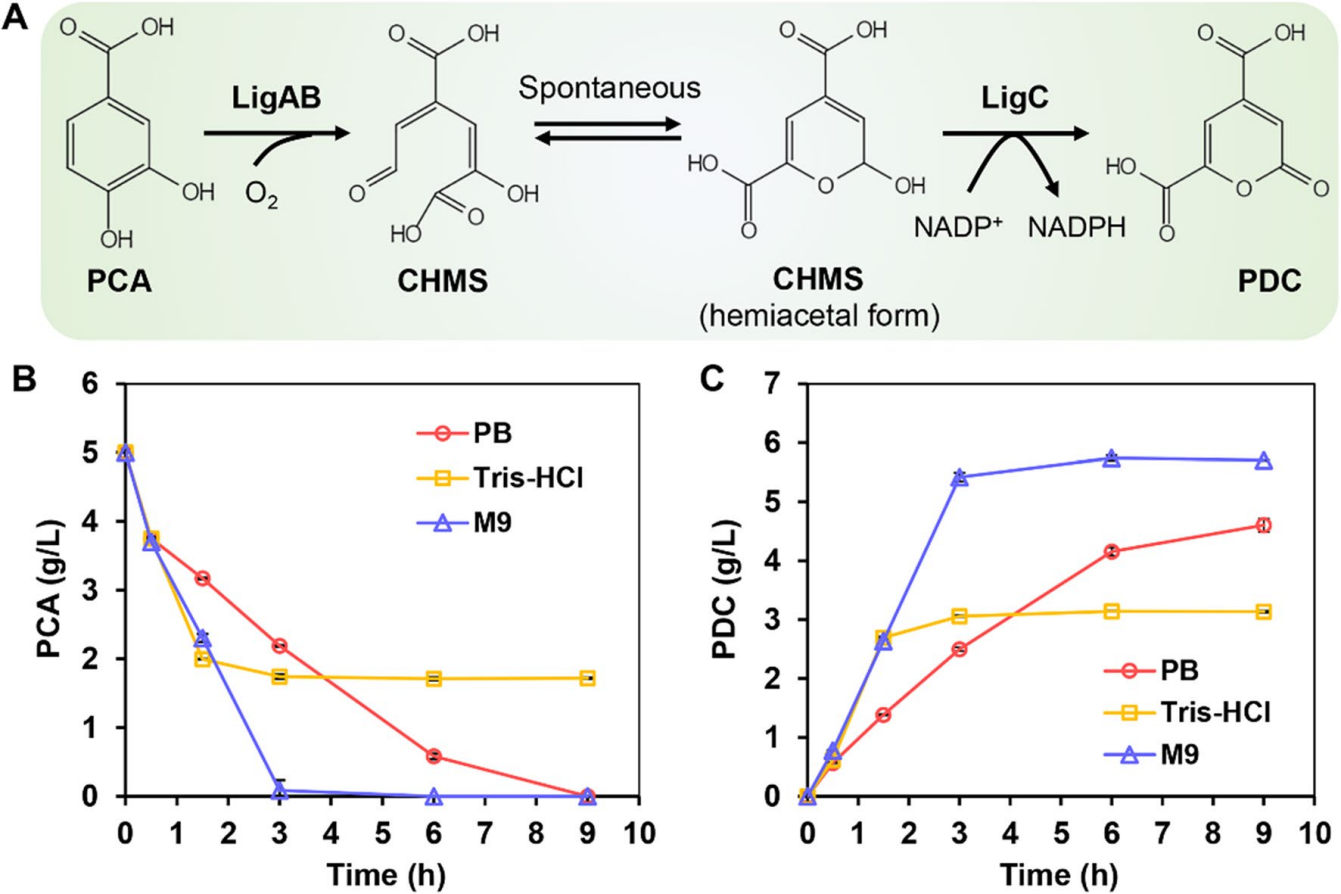
Whole-cell bioconversion of PCA into PDC. **A** Scheme of bioconversion of PCA into PDC catalyzed by LigAB and LigC. Time courses of PCA (**B**) and PDC (**C**) concentrations catalyzed by LigABC biocatalyst in different reaction buffers. The reactions were conducted in 100 mL shake flasks with 10 mL of 100 mM sodium phosphate buffer (PB, pH 7.0) or 100 mM Tris–HCl (pH 7.0) or M9 medium (pH 7.0), 5 OD_600_ of whole-cell biocatalyst, 5 g/L PCA and incubated at 37 °C and 250 rpm. All data and standard errors were derived from three independent biological replicates

In biocatalytic processes, different buffers can significantly affect reaction efficiency. Hence, to achieve efficient whole-cell conversion of PCA into PDC, the effects of different reaction buffers on the biocatalytic processes were first examined. In this case, the bioconversion was achieved with LigAB and LigC (Fig. 1A), both derived from *S. paucimobilis* SYK-6 and widely used in the study of PDC biosynthesis [16, 22, 24, 26]. *E. coli* BL21 (DE3) cells co-expressing the *ligABC* gene cluster were used as whole-cell biocatalysts. Under shaking flask conditions, approximately 98% of PCA was exhausted within 3 h in M9 medium (M9, pH 7.0), and the titer of PDC reached 5.74 g/L after extending the process to 9 h (Fig. 1B, C). The resulting PDC was also analyzed by GC–MS (Additional file 1: Fig. S1). However, when the reaction was performed in 100 mM of sodium phosphate buffer (PB, pH 7.0), the reaction rate and the PDC titer were significantly lower than those of M9 (Fig. 1B, C). Compared with PB, the M9 medium also contains glucose and NH_4_Cl, hence suggesting that adding small amounts of carbon and nitrogen sources could be helpful for maintaining the catalytic activities of whole-cell biocatalysts and improving the substrate conversion ratio. On the other hand, when the reaction was conducted in 100 mM of Tris–HCl buffer (pH 7.0), the reaction rate was comparable to that of M9 for 1.5 h before decreasing rapidly (Fig. 1B, C). This result could be attributed to the small size and the diol-like structure of Tris which could have contributed to its ability to bind and occupy the active site of LigAB, thereby resulting in a mild inhibitory effect [30]. Therefore, for the subsequent experiments, M9 medium was selected as the reaction buffer for the whole-cell bioconversion.

### Screening protocatechuate 4,5-dioxygenase and CHMS dehydrogenase for the efficient conversion of PCA into PDC

**Fig. 2:**
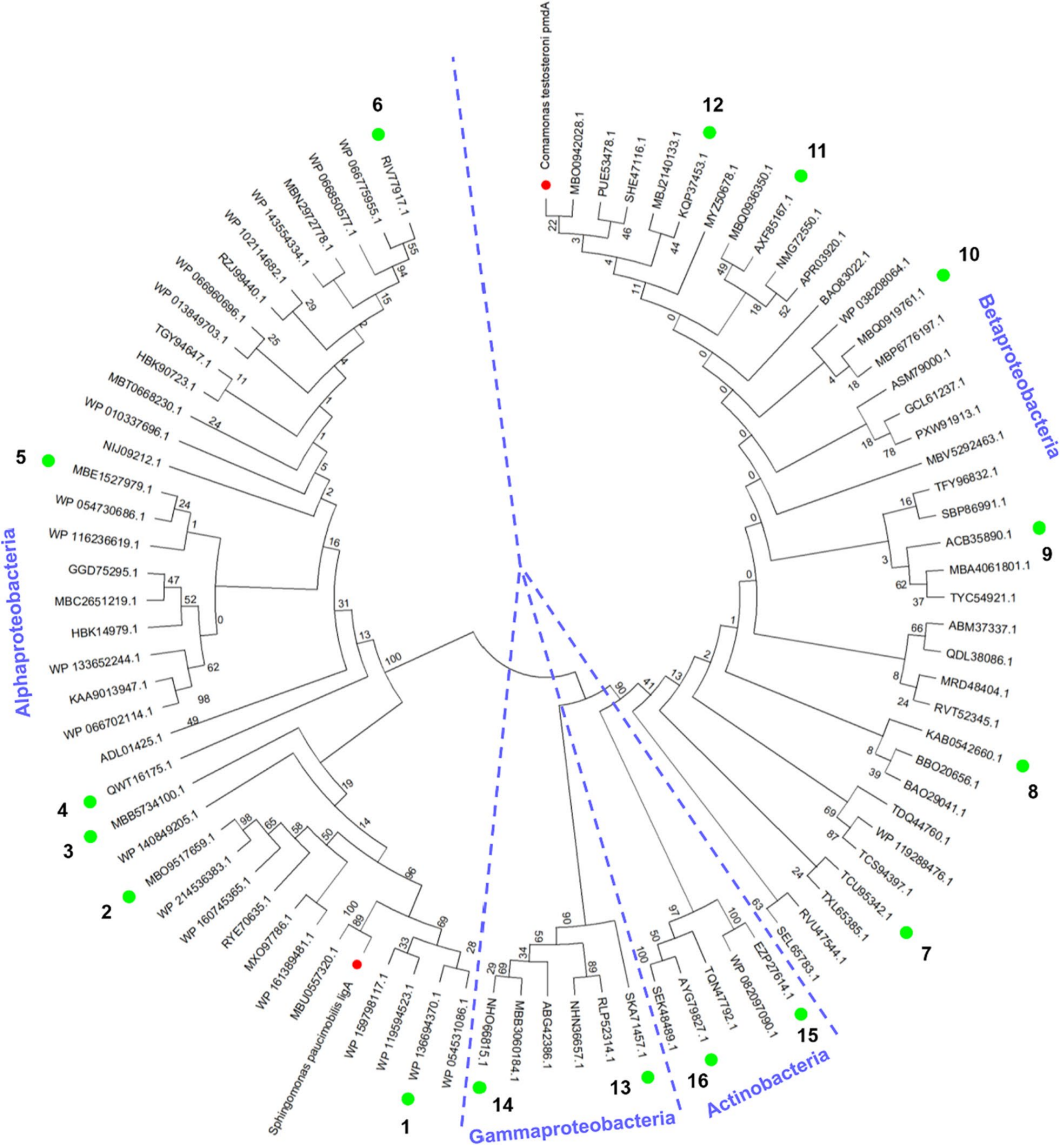
Phylogenetic tree of the protocatechuate 4,5-dioxygenase *α* subunits from different bacteria. A total of 86 sequences derived from *Alphaproteobacteria*, *Betaproteobacteria*, *Gammaproteobacteria* and *Actinobacteria* were selected to draw the phylogenetic tree, and 16 of them (numbered 1–16 and labeled with green circles) were evenly selected for the subsequent studies. The reported LigA and PmdA were labeled with red circles. The percentage of replicate trees in which the associated taxa clustered together in the bootstrap test are shown next to the branches

Based on sequence analysis, the reported *ligABC* [22] and *pmdABC* [31] exist in the form of gene clusters on chromosomes, and therefore, the entire coding sequence of the three genes can be obtained from any one of them. In this case, the protocatechuate 4,5-dioxygenase *α* subunit LigA and PmdA were selected as query sequences for a BLAST search against the protein database of NCBI. The results showed that most of the protocatechuate 4,5-dioxygenases exist in *Alphaproteobacteria* and *Betaproteobacteria*, with a small fraction also present in *Gammaproteobacteria* and *Actinobacteria*. Based on the sequence identities, a total of 86 sequences were selected together with LigA and PmdA to draw the phylogenetic tree, with the number of sequences derived from *Alphaproteobacteria*, *Betaproteobacteria*, *Gammaproteobacteria* and *Actinobacteria* being 38, 37, 6 and 5, respectively. Through phylogenetic analysis, the proteins clustered within their own classes in the tree according to their evolutionary characteristics (Fig. 2), thus indicating that the protocatechuate 4,5-dioxygenases are highly conserved in different clades. A total of 16 sequences (numbered 1–16) were then evenly selected from the tree, and included 1ABC-6ABC from *Alphaproteobacteria*, 7ABC-12ABC from *Betaproteobacteria*, 13ABC and 14ABC from *Gammaproteobacteria* as well as 15ABC and 16ABC from *Actinobacteria* (Fig. 2). By aligning their genome sequences, the entire coding sequences for all ABC gene clusters were finally obtained (Additional file 1: Table S2).

**Fig. 3:**
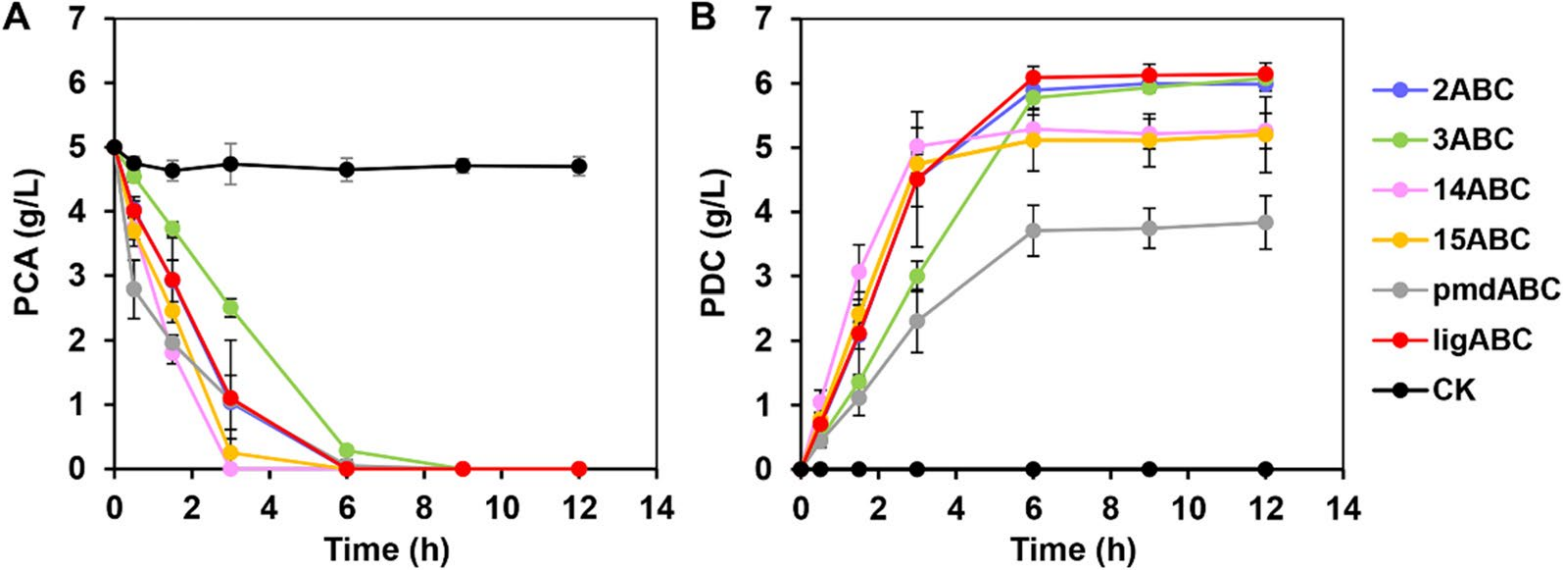
Whole-cell bioconversion of PCA into PDC catalyzed by different ABC whole-cell biocatalysts. Time courses of PCA (**A**) and PDC (**B**) concentrations catalyzed by different biocatalysts. The reactions were conducted in 100 mL shake flasks with 10 mL M9 medium, 5 OD_600_ of whole-cell biocatalyst, 5 g/L PCA and incubated at 37 °C and 250 rpm. *E. coli* BL21(DE3) strain transformed with pRSFDuet-1 plasmid was used as the negative control (CK). All data and standard errors were derived from three independent biological replicates

The genes of *ligAB* and *ligC* derived from different organisms were first cloned into the pRSFDuet-1 plasmid (Additional file 1: Table S2). After that, these plasmids were transformed into *E. coli* BL21 (DE3) cells, and protein expression was carried out in shake flasks to obtain whole-cell biocatalysts that were named 1ABC-16ABC, LigABC and PmdABC. When analyzing the bioconversion processes, these whole-cell biocatalysts displayed differences in their ability to convert PCA into PDC, especially in terms of their conversion ratios and reaction rates (Additional file 1: Table S3). In particular, the PDC titers of 2ABC, 3ABC and LigABC were up to 6.0 g/L with approximately 100% of molar yields at 12 h. In addition, the reaction rate of 2ABC was comparable to that of LigABC, while that of 3ABC was slower compared with LigABC (Fig. 3A, B and Additional file 1: Table S3). Unexpectedly, the reaction rates of 14ABC and 15ABC were significantly faster than that of LigABC, with less than 5% of PCA remaining in the medium at 3 h. However, as the reaction continued, the final titer of PDC was only 5.2 g/L for 14ABC or 15ABC with an 87% molar yield, which was significantly lower than those of LigABC or 2ABC (Fig. 3A, B and Additional file 1: Table S3). This result suggested that only part of the intermediate CHMS generated by 14AB or 15AB was converted into PDC. It has been reported that in addition to being converted into PDC, CHMS can also spontaneously generate 2,4-pyridine dicarboxylic acid through the ammonia cyclisation reaction in the presence of NH_4_Cl [19, 32, 33]. Thus, avoiding the accumulation of intermediate CHMS by slowing down the reaction catalyzed by 14AB or 15AB (protocatechuate 4,5-dioxygenase), or by increasing the activity of 14C or 15C (CHMS dehydrogenase) may be helpful for further improving the conversion ratio.

### Optimizing reaction conditions for efficient conversion of PCA into PDC

**Fig. 4:**
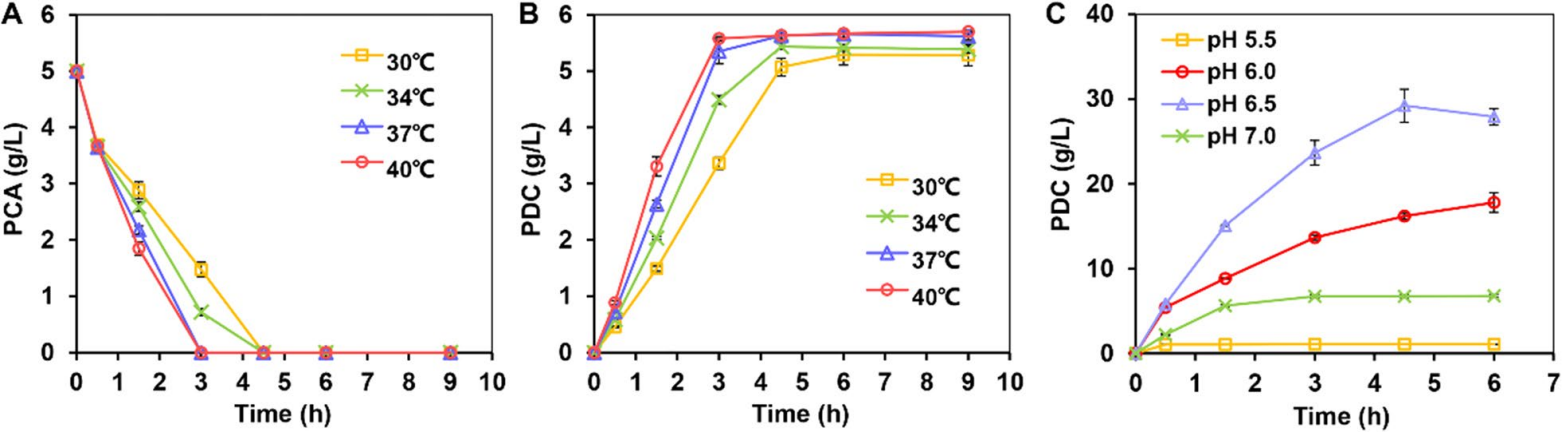
Effects of temperature and pH on whole-cell bioconversion of PCA into PDC. Time courses of PCA (**A**) and PDC (**B**) concentrations catalyzed by 14ABC biocatalyst at different temperatures. The reactions were conducted in 100 mL shake flasks with 10 mL M9 medium, 5 OD_600_ of whole-cell biocatalyst, 5 g/L PCA and incubated at 250 rpm. **C** Time courses of PDC production catalyzed by 14ABC biocatalyst in the pH range of 5.5 to 7.0. The reactions were conducted in 5-L bioreactors at 37 °C with 1 L M9 medium and 30 OD_600_ of whole-cell biocatalyst. Approximately 10 g/L PCA was supplemented into the bioreactor at one time when the DO level rose. All data and standard errors were derived from three independent biological replicates

Changes in temperature and pH can significantly affect the progress of enzymatic reactions. Therefore, these parameters were optimized for the whole-cell biocatalytic reactions. To confirm the optimum temperature, the reaction was performed in the range of 30 °C to 40 °C. With the increase in temperature from 30 to 37 °C, the reaction rate gradually accelerated, but no significant differences in the rates of PCA consumption and PDC synthesis were observed between 37 and 40 °C within 3 h (Fig. 4A, B). Since the higher temperature (40 °C) was not conducive to maintaining cell viability, 37 °C was selected as the optimum temperature for subsequent whole-cell biocatalytic studies.

To confirm the optimum pH, whole-cell biocatalytic reactions were first conducted in the pH range of 5.0 to 8.0 in shake flasks. With increasing pH, the reaction efficiency first increased before subsequently decreasing. In particular, at a pH of 5.5, the fastest reaction rate and highest PDC titer were achieved (Additional file 1: Fig. S2A, B). However, unexpectedly, when the reaction was conducted in a 5-L bioreactor at this pH, a final titer of only 1.07 g/L was obtained for PDC, even though the biocatalyst load reached 30 OD_600_ (Fig. 4C). This discrepancy in outcomes between shake flasks and the bioreactor prompted us to reconfirm the optimum pH for conversion in bioreactors. In this case, when the pH increased from 5.5 to 7.0, the reaction efficiency initially increased before decreasing, as initially observed. However, the optimum pH for the reaction was changed from 5.5 to 6.5, and the final titer of PDC reached 28.39 g/L, with this value being significantly higher than that of other pH conditions (Fig. 4C). In phosphate buffers, the optimum pH for protocatechuate 4,5-dioxygenase and CHMS dehydrogenase was reported to be 7.5 and 8.0, respectively, even though the CHMS dehydrogenase was most stable at pH 6.5–7.0 [30, 34, 35]. These results showed that a pH of 6.5 was more conducive for the whole-cell bioconversion process on a large scale. In biocatalytic process requiring two different catalytic enzymes, individual reaction rates may restrict one another. The best reaction efficiency can, therefore, be achieved only when the activities of the two enzymes are reasonably complemented.

### Highly efficient conversion of PCA into PDC on a large scale

To achieve efficient conversion of PCA into PDC on a large scale, whole-cell bioconversion was performed in a 5-L bioreactor. For this experiment, a fed-batch fermentation was first carried out to prepare enough biocatalysts of 2ABC, 14ABC and LigABC. After induction with 0.2 mM IPTG and subsequent growth for an additional 6 h, the 2ABC, 14ABC and LigABC strains reached an OD_600_ of 61.6, 50.8 and 59.0, respectively (Additional file 1: Fig. S3). By analyzing bioconversion processes, the PDC titers of the fresh biocatalysts were approximately five-fold higher than those obtained from the frozen biocatalysts whenever the reactions were conducted at pH 5.5 or 6.5 (Additional file 1: Fig. S2C). At the end of the fed-batch fermentation, the cells were harvested and directly used as whole-cell biocatalysts for subsequent bioconversion processes.

**Fig. 5:**
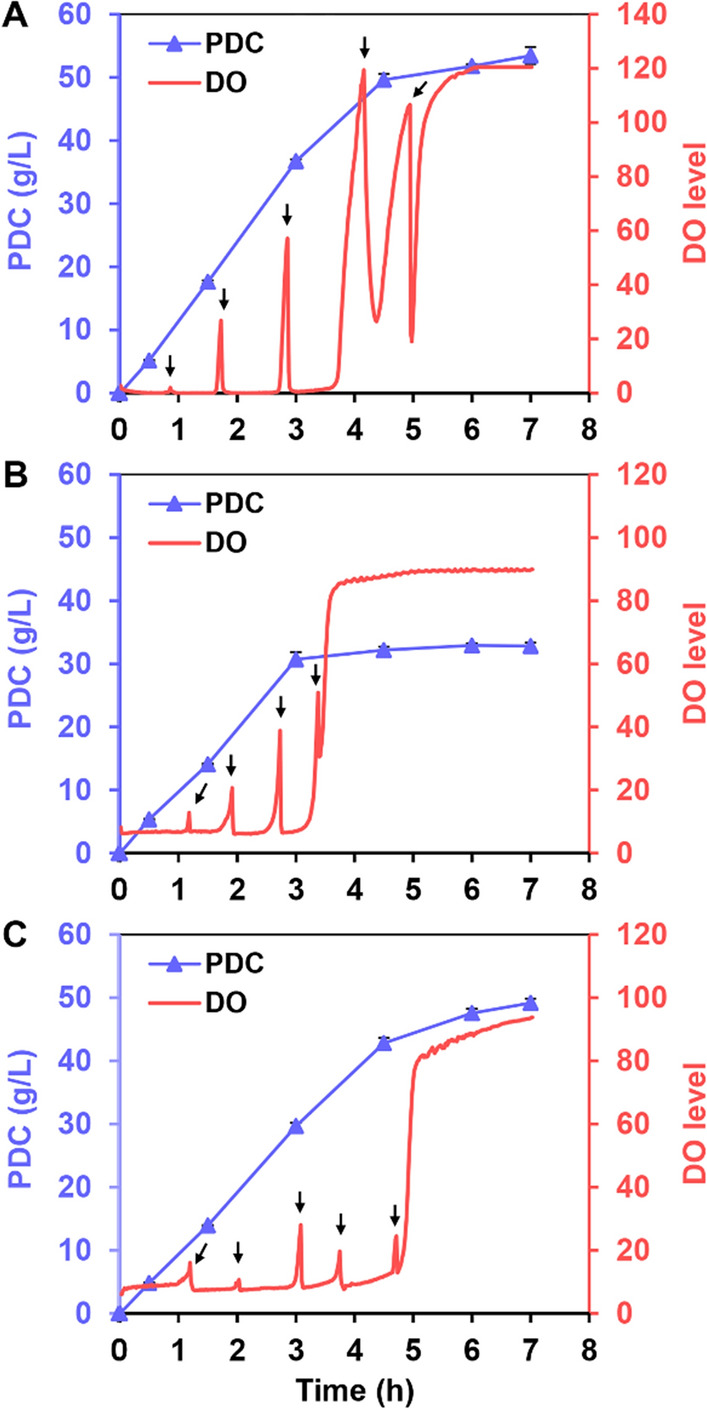
Whole-cell bioconversion of PCA into PDC on a large scale. Time courses of the PDC production catalyzed by the biocatalysts of 2ABC (**A**), 14ABC (**B**) and LigABC (**C**). The reactions were conducted in 5-L bioreactors with 1 L M9 medium and 30 OD_600_ of whole-cell biocatalysts at 37 °C and pH 6.5. Approximately 10 g/L PCA was supplemented into the bioreactor at one time when the DO level rose (indicated with black arrows). The error bars represent the standard deviation of the three replicates, and the DO curve is the result of one run

Since the oxidative ring-opening reaction of PCA, catalyzed by protocatechuate 4,5-dioxygenase, is an O_2_-dependent one [30], the DO level can be used to indicate the residual amount of PCA. In addition, because PCA possesses strong cellular toxicity that hampers both cell growth and intracellular metabolism [36], a batch feeding strategy was adopted to avoid the influence of high concentrations of PCA on the catalytic activity of the whole-cell biocatalysts. In this case, with each rise in DO levels, approximately 10 g/L PCA was supplemented into the bioreactor to maintain the PCA concentration at a relatively low level throughout the bioconversion process (Fig. 5A–C). This strategy of batch feeding the PCA can in fact be crucial for efficient conversions. By analyzing whole-cell bioconversion processes, 2ABC was significantly better than 14ABC and LigABC in terms of both reaction rate and PDC titer. When excess PCA was provided, a final PDC titer of 53.46 g/L and a molar yield of 95.2% were obtained for 2ABC with a productivity of 7.64 g/L/h (Fig. 5A). Under similar conditions, the PDC titer for LigABC was only 49.18 g/L, which was 8.7% lower than that of 2ABC, while the productivity was 7.03 g/L/h (Fig. 5C). The reaction rate of 14ABC was comparable to that of LigABC within 3 h, but it subsequently decreased rapidly to reach a final PDC titer of only 32.83 g/L, a molar yield of 75.8% as well as a productivity of 4.69 g/L/h (Fig. 5B). These results showed that 2ABC was the most efficient whole-cell biocatalyst for the conversion of PCA into PDC on a large scale.

### Efficient multi-step biosynthesis of PDC from glucose

**Fig. 6:**
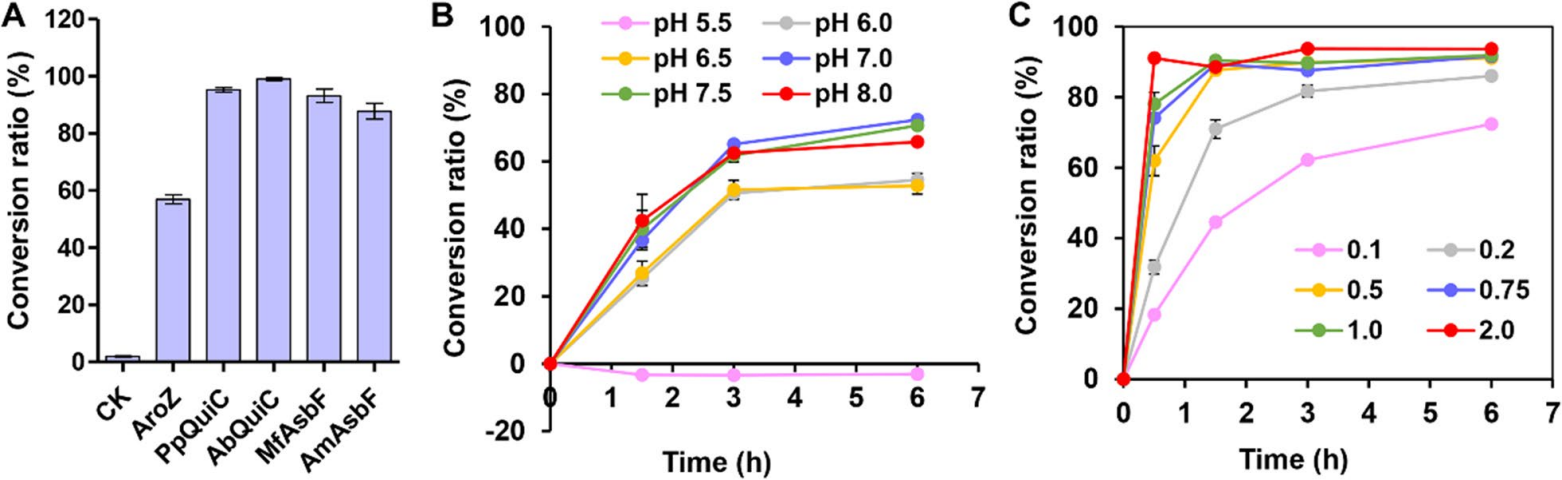
Whole-cell bioconversion of DHS into PCA. **A** Bioconversion of DHS into PCA catalyzed by 3 OD_600_ of biocatalysts expressing different 3-dehydroshikimate dehydratases. *E. coli* BL21(DE3) strain transformed with pET-30a plasmid was used as the negative control (CK). **B** Time courses of the conversion ratio catalyzed by 0.2 OD_600_ of AbQuiC biocatalyst in the pH range of 5.5 to 8.0. **C** Time courses of the conversion ratio catalyzed by AbQuiC biocatalyst with different loads at pH 7.0. The reactions were conducted in 100 mL shake flasks with 60 g/L of DHS and incubated at 37 °C and 250 rpm. All data and standard errors were derived from three independent biological replicates

Although efficient production of PDC has been achieved using pure PCA as substrate, it is economically unfeasible for industrial scale production due to the high costs associated with PCA. Alternatively, the use of cheap glucose as substrate could significantly reduce the production cost. Since PCA possesses strong cellular toxicity [36], it is difficult for *E. coli* to produce high titers of this compound directly from glucose. In our previous work, a DHS-overproducing *E. coli* strain WJ060 was successfully constructed through systematic metabolic engineering [29]. Tto achieve efficient whole-cell bioconversion of DHS into PCA, the reaction efficiencies of five different 3-dehydroshikimate dehydratases, derived from *Klebsiella pneumoniae* (AroZ) [37], *P. putida* GB-1 (PpQuiC), *Acinetobacter baylyi* ADP1 (AbQuiC), *Microbacterium foliorum* (MfAsbF) and *Alteromonas macleodii* (AmAsbF), were then compared and analyzed. Using DHS broth as the substrate, the cells expressing the above 3-dehydroshikimate dehydratases could efficiently convert DHS into PCA, with those expressing AbQuiC exhibiting the highest catalytic efficiency (Fig. 6A). Indeed, with a conversion ratio of 99.0% (mol/mol), AbQuiC was selected for whole-cell bioconversion in follow-up studies.

Subsequently, the optimum pH and biocatalyst load for the above reaction was determined. It has been reported that 3-dehydroshikimate dehydratase has an optimal pH within alkaline range [38–40]. In this study, the cells expressing AbQuiC exhibited the highest reaction efficiency at pH 7.0 and pH 7.5, while at a pH lower than 6.5, the conversion ratio decreased significantly (Fig. 6B). Given that DHS is chemically unstable under alkaline conditions, the subsequent whole-cell bioconversion for AbQuiC was, therefore, carried out at pH 7.0. The amount of whole-cell biocatalysts loaded into the reaction is also an important factor that influences reaction efficiency and cost feasibility. With an increase in the biocatalyst load (0.1 to 2.0 OD_600_), the reaction rate increased gradually, with a molar yield of 91.1% obtained when AbQuiC cells had an OD_600_ of 2.0. However, when the reaction time was extended to 6 h, the conversion ratio of cells with an OD_600_ of 0.5 was also equal to that of cells with an OD_600_ of 2.0 (Fig. 6C).

**Fig. 7:**
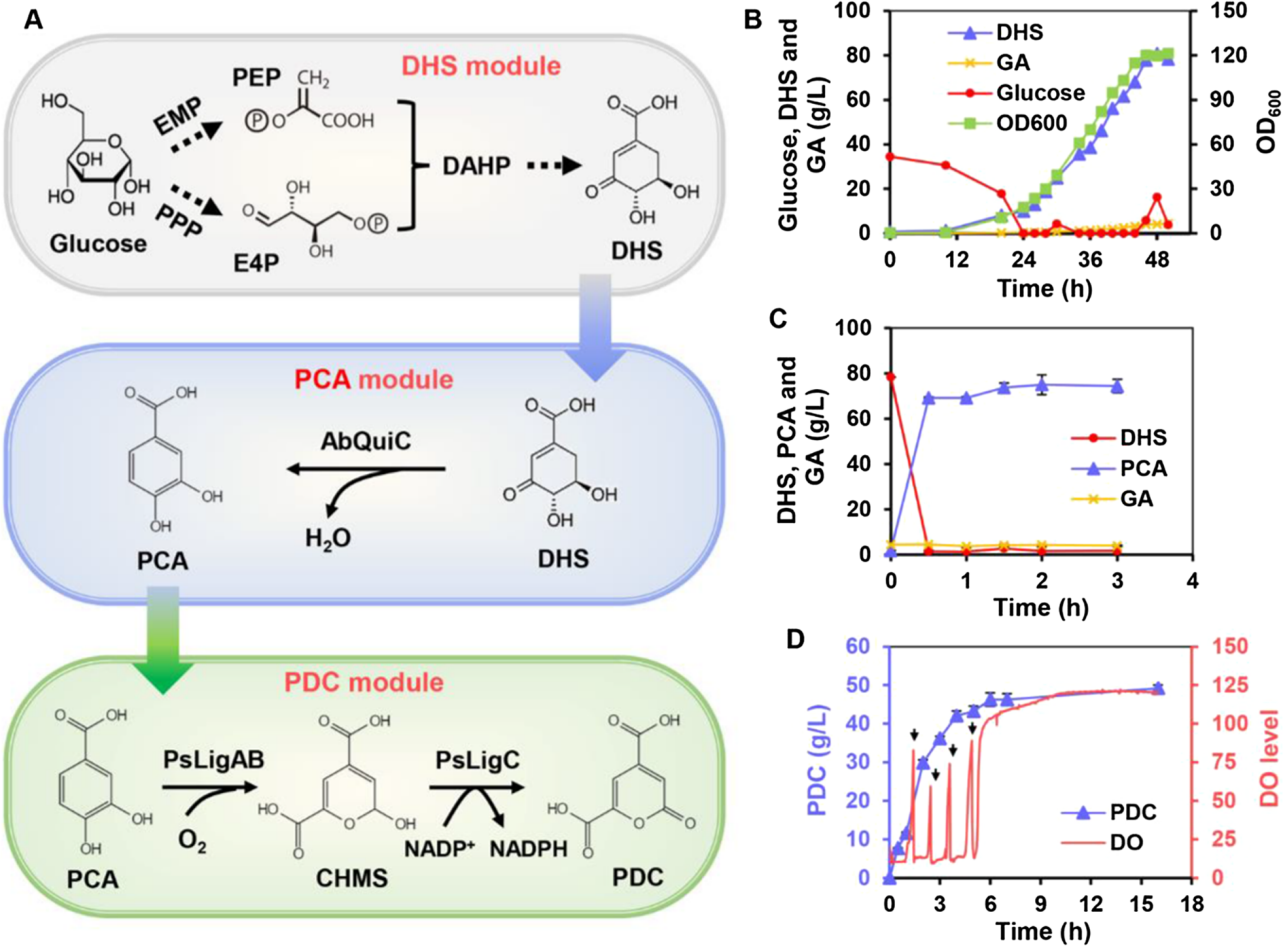
Multi-step biosynthesis of PDC from glucose. **A** Scheme of the multi-step biosynthesis of PDC from glucose. *EMP* Embden–Meyerhof–Parnas pathway, *PPP* pentose phosphate pathway, *PEP* phosphoenolpyruvate, *E4P* d-erythrose 4-phosphate, *DAHP* 3-deoxy-d-arabino-heptulosonate-7-phosphate. **B** Biosynthesis of DHS from glucose by the WJ060 strain through fed-batch fermentation. **C** Bioconversion of fermentative DHS into PCA catalyzed by 2 OD_600_ of AbQuiC biocatalyst. The reaction was conducted in a 5-L bioreactor at 37 °C and pH 7.0. **D** Bioconversion of the PCA broth into PDC catalyzed by 2ABC (PsLigABC) biocatalyst. The reaction was conducted in a 5-L bioreactor with ~ 30 OD_600_ of whole-cell biocatalyst at 37 °C and pH 6.5. Approximately 15 g/L PCA was supplemented into the bioreactor at one time when the DO level rose (indicated with black arrows). The error bars represent the standard deviation of the three replicates, and the DO curve is the result of one run

Based on the above results, the PDC biosynthetic pathway was divided into three synthetic modules, namely the DHS module, the PCA module and the PDC module, all of which account for the bioconversion of glucose to DHS, DHS to PCA and PCA to PDC, respectively (Fig. 7A). First, the rationally designed WJ060 strain was grown in minimal medium, and through fed-batch fermentation, it produced 78.28 g/L of DHS from glucose with a molar yield of 31.2%. In addition to DHS, a small amount of by-product GA (4.37 g/L) was also detected in the fermentation broth (Fig. 7B). Second, the high concentration of DHS (78.28 g/L) in the broth was effectively converted into PCA (74.35 g/L) at pH 7.0 using AbQuiC biocatalyst with an OD_600_ of 2.0. During the whole bioconversion process, the amount of GA in the fermentation broth did not change significantly (Fig. 7C). Finally, the PCA broth was directly used as substrate to produce PDC using the batch feeding strategy. In this case, the conversion of bio-based PCA into PDC was conducted in a bioreactor using cells expressing 2ABC (named PsLigABC) as the biocatalyst. After bioconversion for 16 h, the final PDC titer reached 49.19 g/L with a molar yield of 101.1% (Fig. 7D). When calculating the production of PDC using glucose as the substrate, the molar conversion ratio was up to 27.2%. In addition to PCA, the protocatechuate 4,5-dioxygenase can also catalyze the oxidative ring-opening of GA to generate 4-oxalomesaconate (OMA), with the latter’s further dehydration into PDC catalyzed by PDC hydrolase (LigI) [18, 41]. The GA by-product in the fermentation broth was also completely consumed, suggesting that GA might be converted into PDC, catalyzed by PsligAB and other intracellular hydrolases with similar functions to LigI, to achieve a molar yield of higher than 100%. If the conversion of the GA by-product was also taken into account, the molar yield decreased to 95.8% (PCA + GA).

In this study, the efficient biosynthesis of PDC was achieved using a well-designed whole-cell biocatalytic system. When pure PCA was used as substrate, the 2ABC biocatalyst produced 53.46 g/L PDC within 7 h, with a molar yield of 95.2% (Fig. 5A and Table 1). Johnson et al. [19] used engineered *P. putida* as the biocatalysts and achieved the highest PDC titer (58 g/L) reported to date from 4-hydroxybenzoate, with a molar yield of 80.7%. However, the conversion time was as long as 288 h. When glucose was used as substrate, a final PDC titer of 49.19 g/L was achieved using an optimized multi-step biosynthesis system, and interestingly, this represented the highest titer for PDC production from glucose reported to date (Fig. 7 and Table 1). However, due to the low conversion ratio of the DHS module (31.2%), the final molar yield from glucose was only 27.2%. It is, therefore, speculated that a one-step production of PDC from glucose in subsequent studies would yield a significantly improved conversion ratio.

**Table 1:** Production of PDC by engineered strains

Strain	Substrate	Cultivation	Titer (g/L)	Yield	Time (h)	References
*P. putida* PpY1100 with pDVABC	PCA	5-L bioreactor; fed-batch	~ 11	–	36	[22]
*E. coli* BL21(DE3) with pRSF-2ABC	PCA	5-L bioreactor; fed-batch	53.46	0.952 mol/mol	22 (15 + 7)	This study
*P. putida* KT2440 Δ*pcaGH*Δ*crc* with pSEVA631-ligABC	*p*-Coumarate	5-L bioreactor; fed-batch	22.7	1.0 mol/mol	110	[16]
*N. aromaticivorans* DSM12444 Δ*ligI*Δ*desCD*	Vanillin and vanillate	0.25-L bioreactor; fed-batch	4.9	0.103 mol/mol	48	[20]
*P. putida* KT2440 Δ*pcaHG*::Ptac:*ligABC* (CJ251)	4-Hydroxybenzoate	2.5-L bioreactor; fed-batch	58	0.81 mol/mol	288	[19]
*P. putida* PpY1100 with pVapoligVABC	Vanillin and vanillate derived from lignin	Bioreactor; fed-batch	9.21 (50 mM)	–	18	[23]
*P. putida* PpY1100-dHG with pJFVV2AB and pDVZ21X	Bamboo extract	1-L bioreactor; fed-batch	8.7	0.936 mol/mol	24	[42]
*P. putida* KT2440 KT2440 Δ*pcaHG*::Ptac:*ligABC* Δ*vanAB*::Ptac:*vanAB*_HR199_	Equimolar mixture of syringate, *p*-coumarate, and ferulate	Shake flask	0.79 (4.3 mM)	0.93 mol/mol	24	[21]
*E. coli* PCA strain and *E. coli* PDC_PCA_ strain	Terephthalic acid derived from PET waste	Shake flask	0.57 (3.11 mM)	0.99 mol/mol	6	[24]
*E. coli* BL21(DE3) harboring three plasmids (pACYC-aroF^fbr^-aroB, pCDF-ubiC-pobA, and pFT-ligABC-qutC)	Algae hydrolysate and glucose	Shake flask	1.22	0.161	24	[25]
*E. coli* GYT1 with pTacFABC and pBBR1G^fbr^-EcA	Glucose	6.6-L bioreactor; fed-batch	16.72	0.201 g/g	96	[27]
*E. coli* WJ060, *E. coli* BL21(DE3) with pET30a-AbquiC and *E. coli* BL21(DE3) with pRSF-2ABC	Glucose	5-L bioreactor; fed-batch	49.19	0.272 mol/mol	66	This study

## Conclusions

In this study, an efficient multi-step biosynthesis method for the microbial production of PDC was demonstrated by smoothly integrating a DHS module, a PCA module and a PDC module. The final PDC titer achieved was 49.19 g/L with a molar yield of 27.2%, which represented the highest titer for PDC production from glucose reported to date. In addition, this work also provides several effective enzymes for the microbial biosynthesis of PDC. Although an efficient multi-step biosynthesis of PDC with a high titer has been achieved, the microbial production process tends to be cumbersome. Future studies in this direction can, therefore, focus on the application of metabolic engineering technologies in an attempt to construct PDC-overproducing *E. coli* cell factories based on the WJ060 strain as well as the above effective enzymes, especially in view of achieving a one-step production of PDC with higher titers or yields from glucose. Besides, if a higher PDC biosynthesis rate or titer could be achieved, the rate of PCA uptake or PDC efflux could become a new limiting factor. Therefore, the mining PCA- and PDC-specific transporters could be of great significance to the construction of efficient microbial cell factories.

## Supplementary Information

**Additional file 1: Figure S1.** Identification of PDC product by GC–MS. **Figure S2.** The effects of pH and biocatalyst on whole-cell bioconversion of PCA into PDC. **Figure S3.** Preparation of whole-cell biocatalysts by fed-batch fermentation. **Table S1.** The strains and plasmids used in this study. **Table S2.** The GenBank accession numbers of the protocatechuate 4,5-dioxygenases and the CHMS dehydrogenases used in this study. **Table S3.** Time course of PDC production from PCA catalyzed by different whole-cell biocatalysts.

## Data Availability

All data generated or analyzed during this study are included in this published article and its supplementary information files.
