# Supplementary material for: Multi-step biosynthesis of the biodegradable polyester monomer 2-pyrone-4,6-dicarboxylic acid from glucose
Source: Biotechnol Biofuels Bioprod. 2023 Jun 1;16:92. doi: 10.1186/s13068-023-02350-y (PMC10233912; doi:10.1186/s13068-023-02350-y)
Supplement: Supplementary file 1 — Additional file 1: Figure S1. Identification of PDC product by GC–MS. Figure S2. The effects of pH and biocatalyst on whole-cell bioconversion of PCA into PDC. Figure S3. Preparation of whole-cell biocatalysts by fed-batch fermentation. Table S1. The strains and plasmids used in this study. Table S2. The GenBank accession numbers of the protocatechuate 4,5-dioxygenases and the CHMS dehydrogenases used in this study. Table S3. Time course of PDC production from PCA catalyzed by different whole-cell biocatalysts. [file 13068_2023_2350_MOESM1_ESM.docx]

**Multi-step biosynthesis of the** **biodegradable polyester monomer 2-pyrone-4,6-dicarboxylic acid** **from glucose**

Dan Zhou^1,2,3†^, Fengli Wu^2,3†*^, Yanfeng Peng^2,3^, Muneer Ahmed Qazi^2,3,4^, Ruosong Li^2,3,5^, Yongzhong Wang^1*^, Qinhong Wang^2,3^

^1^Key Laboratory of Biorheological Science and Technology (Chongqing University), Ministry of Education, College of Bioengineering, Chongqing University, Chongqing 400030, China

^2^Tianjin Institute of Industrial Biotechnology, Chinese Academy of Sciences, Tianjin 300308, China

^3^National Center of Technology Innovation for Synthetic Biology, Tianjin 300308, China

^4^Institute of Microbiology, Faculty of Natural Science, Shah Abdul Latif University, Khairpur, 66020 Sindh, Pakistan

^5^College of Biotechnology, Tianjin University of Science & Technology, Tianjin 300457, China

^†^Dan Zhou and Fengli Wu have contributed equally to this work

^*^Correspondence: wu_fl@tib.cas.cn; wangyzh@cqu.edu.cn

**Additional Figures**

**Figure S1** Identification of PDC product by GC-MS. Gas chromatogram of the trimethylsilylated (TMS) derivative of authentic PDC compound (**A**) and of PDC produced in flask culture (**C**). Mass spectrum of the TMS derivative of authentic PDC compound (**B**) and of PDC produced in flask culture (**D**).

**Figure S2** The effects of pH and biocatalyst on whole-cell bioconversion of PCA into PDC. Time courses of PCA (**A**) and PDC (**B**) concentrations catalyzed by 14ABC biocatalyst in the pH range of 5.0 to 8.0. The reactions were conducted in 100 mL shake flasks with 10 mL 100 mM sodium phosphate buffer (pH 5.0 to 8.0), 1 OD_600_ of whole-cell biocatalyst, 1 g/L PCA and incubated at 37 ℃ and 250 rpm. **C** The titers of PDC catalyzed by frozen or fresh 14ABC biocatalyst at 0.5 h. The reaction was conducted in a 5-L bioreactor at 37 ℃ with 1 L M9 medium, 10 g/L of PCA and 30 OD_600_ of whole-cell biocatalyst. Data are presented as the mean ± standard deviation of three independent experiments. ** p < 0.01.

**Figure S3** Preparation of whole-cell biocatalysts by fed-batch fermentation. Time courses of cell growth (OD_600_ with green squares) and glycerol concentration (orange circles) for 2ABC (**A**), 14ABC (**B**) and LigABC (**C**) strains. The fed-batch fermentation for each strain was conducted at least three times in a 5-L bioreactor, and the data shown in the figure are the results of one run.

**Additional Tables**

**Table S1** The strains and plasmids used in this study.

| **Name** | **Characteristics** | **Source** |
| --- | --- | --- |
| **Strains** | | |
| *E. coli* DH5α | F^-^φ80 *lacZ*ΔM15 Δ(*lac*ZYA-*arg*F) U169 *end*A1 *rec*A1 *hsd*R17(rk^-^, mk^+^) *sup*E44λ^-^ *thi*^-^1 *gyr*A96 *rel*A1 *pho*A | TransGen Biotech |
| *E. coli* BL21(DE3) | F^-^ *ompT* *hsdS_B_* (*r_B_*^-^ *m_B_*^-^) *gal* *dcm* (DE3) | Novagen |
| WJ060 | DSM 1576 P1-*aroE*^TTG^ P2-*aroF*^fbr^ Δ*tyrR* P4-*tktA* Δ*ptsI* P1-*galP* P4-*glk* P1-*pykF*^TTG^ P1-*pykA*^TTG^ P1-*pgi*^TTG^ | [29] |
| **Plasmids*** | | |
| pRSFDuet-1 | *RSF1030* *kan lacI* | Novagen |
| pRSF-ligABC | *RSF1030* *kan lacI* P*_T7_*-*ligA*-RBS-*ligB* P*_T7_*-*ligC* | This study |
| pRSF-pmdABC | *RSF1030* *kan lacI* P*_T7_*-*pmdA*-RBS-*pmdB* P*_T7_*-*pmdC* | This study |
| pRSF-1ABC | *RSF1030* *kan lacI* P*_T7_*-*1A*-RBS-*1B* P*_T7_*-*1C* | This study |
| pRSF-2ABC | *RSF1030* *kan lacI* P*_T7_*-*2A*-RBS-*2B* P*_T7_*-*2C* | This study |
| pRSF-3ABC | *RSF1030* *kan lacI* P*_T7_*-*3A*-RBS-*3B* P*_T7_*-*3C* | This study |
| pRSF-4ABC | *RSF1030* *kan lacI* P*_T7_*-*4A*-RBS-*4B* P*_T7_*-*4C* | This study |
| pRSF-5ABC | *RSF1030* *kan lacI* P*_T7_*-*5A*-RBS-*5B* P*_T7_*-*5C* | This study |
| pRSF-6ABC | *RSF1030* *kan lacI* P*_T7_*-*6A*-RBS-*6B* P*_T7_*-*6C* | This study |
| pRSF-7ABC | *RSF1030* *kan lacI* P*_T7_*-*7A*-RBS-*7B* P*_T7_*-*7C* | This study |
| pRSF-8ABC | *RSF1030* *kan lacI* P*_T7_*-*8A*-RBS-*8B* P*_T7_*-*8C* | This study |
| pRSF-9ABC | *RSF1030* *kan lacI* P*_T7_*-*9A*-RBS-*9B* P*_T7_*-*9C* | This study |
| pRSF-10ABC | *RSF1030* *kan lacI* P*_T7_*-*10A*-RBS-*10B* P*_T7_*-*10C* | This study |
| pRSF-11ABC | *RSF1030* *kan lacI* P*_T7_*-*11A*-RBS-*11B* P*_T7_*-*11C* | This study |
| pRSF-12ABC | *RSF1030* *kan lacI* P*_T7_*-*12A*-RBS-*12B* P*_T7_*-*12C* | This study |
| pRSF-13ABC | *RSF1030* *kan lacI* P*_T7_*-*13A*-RBS-*13B* P*_T7_*-*13C* | This study |
| pRSF-14ABC | *RSF1030* *kan lacI* P*_T7_*-*14A*-RBS-*14B* P*_T7_*-*14C* | This study |
| pRSF-15ABC | *RSF1030* *kan lacI* P*_T7_*-*15A*-RBS-*15B* P*_T7_*-*15C* | This study |
| pRSF-16ABC | *RSF1030* *kan lacI* P*_T7_*-*16A*-RBS-*16B* P*_T7_*-*16C* | This study |
| pET30a | *pBR322 kan lacI* | Novagen |
| pET30a-aroZ | *pBR322 kan lacI* P*_T7_*-*aroZ* | This study |
| pET30a-PpquiC | *pBR322 kan lacI* P*_T7_*-*PpquiC* | This study |
| pET30a-AbquiC | *pBR322 kan lacI* P*_T7_*-*AbquiC* | This study |
| pET30a-MfasbF | *pBR322 kan lacI* P*_T7_*-*MfasbF* | This study |
| pET30a-AmasbF | *pBR322 kan lacI* P*_T7_*-*AmasbF* | This study |

*****The plasmids were transformed into *E. coli* BL21(DE3) cells to obtain the corresponding protein expression strains.

**Table S2** The GenBank accession numbers of the protocatechuate 4,5-dioxygenases and the CHMS dehydrogenases used in this study.

| **Gene** | **Organism** | **Nucleotide accession no.** | **Protocatechuate 4,5-dioxygenase *α* and *β* subunit (A/B) accession no.** | **CHMS dehydrogenase (C) accession no.** |
| --- | --- | --- | --- | --- |
| ligABC | *Sphingomonas paucimobilis* SYK-6 | AB073227.1 | BAB88742.1/BAB88743.1 | BAB88744.1 |
| pmdABC | *Comamonas testosteroni* BR6020 | AF305325.1 | AAK73572.1/AAK73573.1 | AAK73574.1 |
| 1ABC | *Alteraurantiacibacter aquimixticola* SSKS-13 | SSHH01000004.1 | TIX48797.1/TIX48798.1 | TIX48799.1 |
| 2ABC | *Porphyrobacter* sp. BIN49 | JAGIBN010000003.1 | MBO9517659.1/MBO9517658.1 | MBO9517657.1 |
| 3ABC | *Altererythrobacter atlanticus* DSM 100738 | JACIJL010000010.1 | MBB5734100.1/MBB5734099.1 | MBB5734098.1 |
| 4ABC | *Sphingobium xenophagum* PH3-15 | CP076557.1 | QWT16175.1/QWT16174.1 | QWT16173.1 |
| 5ABC | *Sphingopyxis* sp. OAS728 | JADBDT010000001.1 | MBE1527979.1/MBE1527978.1 | MBE1527977.1 |
| 6ABC | *Pelagerythrobacter aerophilus* Ery1 | QXFK01000016.1 | RIV77917.1/RIV77918.1 | RIV77919.1 |
| 7ABC | *Pelomonas saccharophila* DSM 654 | SMBU01000015.1 | TCU95342.1/TCU95343.1 | TCU95344.1 |
| 8ABC | *Kerstersia gyiorum* CCUG 47000 | VZPC01000007.1 | KAB0542660.1/KAB0542661.1 | KAB0542662.1 |
| 9ABC | *Leptothrix cholodnii* SP-6 | CP001013.1 | ACB35890.1/ACB35891.1 | ACB35892.1 |
| 10ABC | *Hydrogenophaga aromaticivorans* D2P3 | JAGPWB010000023.1 | MBQ0919761.1/MBQ0919762.1 | MBQ0919763.1 |
| 11ABC | *Ephemeroptericola cinctiostellae* F02 | CP031124.1 | AXF85167.1/AXF85168.1 | AXF85169.1 |
| 12ABC | *Pseudorhodoferax* sp. Leaf274 | LMNA01000027.1 | KQP37453.1/KQP37452.1 | KQP37451.1 |
| 13ABC | *Thiothrix eikelboomii* ATCC 49788 | FUYB01000003.1 | SKA71457.1/SKA71463.1 | SKA71468.1 |
| 14ABC | *Aestuariicella hydrocarbonica* JCM 30134 | JAAONZ010000012.1 | NHO66815.1/NHO66816.1 | NHO66817.1 |
| 15ABC | *Microbacterium oleivorans* RIT293 | JFYO01000005.1 | EZP27614.1 | EZP27613.1 |
| 16ABC | *Streptomyces hundungensis* BH38 | CP032698.1 | AYG79827.1 | AYG79828.1 |

**Table S3** Time course of PDC production from PCA catalyzed by different whole-cell biocatalysts.

| **Biocatalyst** | **PDC titer (g/L)** | | | | | | |
| --- | --- | --- | --- | --- | --- | --- | --- |
|  | **0.0 h** | **0.5 h** | **1.5 h** | **3.0 h** | **6.0 h** | **9.0 h** | **12.0 h** |
| 1ABC | 0.00±0.00 | 0.41±0.01 | 1.04±0.03 | 2.19±0.10 | 4.13±0.04 | 4.85±0.02 | 5.34±0.05 |
| 2ABC | 0.00±0.00 | 0.67±0.07 | 2.09±0.22 | 4.51±0.42 | 5.89±0.05 | 6.00±0.08 | 5.99±0.10 |
| 3ABC | 0.00±0.00 | 0.48±0.03 | 1.36±0.08 | 3.00±0.24 | 5.78±0.17 | 5.93±0.07 | 6.08±0.06 |
| 4ABC | 0.00±0.00 | 0.40±0.02 | 1.06±0.10 | 2.32±0.21 | 4.83±0.44 | 6.23±0.04 | 6.39±0.17 |
| 5ABC | 0.00±0.00 | 0.54±0.04 | 1.50±0.13 | 3.13±0.27 | 5.31±0.19 | 5.60±0.09 | 5.68±0.21 |
| 6ABC | 0.00±0.00 | 0.50±0.12 | 1.12±0.15 | 1.74±0.17 | 2.07±0.24 | 2.11±0.19 | 2.16±0.20 |
| 7ABC | 0.00±0.00 | 0.35±0.02 | 0.84±0.06 | 1.75±0.14 | 3.57±0.21 | 5.29±0.25 | 5.82±0.11 |
| 8ABC | 0.00±0.00 | 0.44±0.14 | 1.36±0.37 | 2.63±0.48 | 3.83±0.56 | 3.89±0.44 | 3.97±0.58 |
| 9ABC | 0.00±0.00 | 0.38±0.01 | 1.03±0.05 | 2.17±0.08 | 4.92±0.22 | 5.91±0.05 | 6.00±0.08 |
| 10ABC | 0.00±0.00 | 0.41±0.05 | 1.22±0.13 | 2.62±0.18 | 4.30±0.39 | 4.37±0.31 | 4.42±0.42 |
| 11ABC | 0.00±0.00 | 0.48±0.09 | 1.24±0.21 | 2.41±0.41 | 4.17±0.46 | 4.60±0.59 | 4.82±0.83 |
| 12ABC | 0.00±0.00 | 0.38±0.06 | 1.03±0.12 | 2.14±0.12 | 4.60±0.25 | 6.10±0.19 | 6.31±0.14 |
| 13ABC | 0.00±0.00 | 0.37±0.01 | 1.06±0.03 | 2.11±0.08 | 3.80±0.16 | 4.40±0.27 | 4.76±0.41 |
| 14ABC | 0.00±0.00 | 1.05±0.19 | 3.06±0.42 | 5.02±0.29 | 5.29±0.22 | 5.21±0.23 | 5.26±0.27 |
| 15ABC | 0.00±0.00 | 0.78±0.01 | 2.42±0.14 | 4.75±0.15 | 5.11±0.48 | 5.11±0.41 | 5.20±0.59 |
| 16ABC | 0.00±0.00 | 0.55±0.18 | 0.86±0.07 | 1.45±0.19 | 2.11±0.37 | 2.34±0.41 | 2.48±0.40 |
| PmdABC | 0.00±0.00 | 0.43±0.10 | 1.11±0.27 | 2.31±0.49 | 3.71±0.39 | 3.74±0.31 | 3.83±0.41 |
| LigABC | 0.00±0.00 | 0.70±0.19 | 2.11±0.64 | 4.51±1.05 | 6.09±0.18 | 6.12±0.17 | 6.15±0.17 |
| CK | 0.00±0.00 | 0.00±0.00 | 0.00±0.00 | 0.00±0.00 | 0.00±0.00 | 0.00±0.00 | 0.00±0.00 |
